# Improving diabetes management in older adults with cancer: a quality improvement project to enhance monitoring during steroid-based systemic anti-cancer therapy

**DOI:** 10.1007/s41999-026-01585-w

**Published:** 2026-09-03

**Authors:** Evelyne Liuu, Rose Evans, Nathanael Bassas-Letissier, Gitanjali Amaratunga, Sarah Compton, Alexandros Georgiou, Tania Kalsi, Yuk-Fun Liu, James Maguire, Clare Nottage, Hannah L. Rush, Carly Welch

**Affiliations:** 1 Department of Ageing and Health, St Thomas’ Hospital, 9th Floor North WingWestminster Bridge, London, SE1 7EH UK; 2 Department of Twin Research, Ageing, & Genetic Epidemiology, King’s College London, 3rd Floor D Block South Wing, St Thomas’ Campus, London, SE1 7EH UK; 3 Department of geriatricsINSERM 1402Centre d’Investigation Clinique, Laboratoire MOVE, University of Poitiers, CHU Poitiers, 2 rue de la Miletrie, 86000 Poitiers, France; 4 King’s College Hospital NHS Foundation Trust, London, UK; 5 Medical Oncology, School of Cancer and Pharmaceutical Sciences, Guy’s and St Thomas’ Hospital, King’s College London, London, UK; 6 Faculty of Life Sciences & Medicine, CARICE, King’s College London, London, UK; 7 Department of Diabetes and Endocrinology, Guy’s and St Thomas’ NHS Foundation Trust, London, UK; 8 Department of Geriatrics, Poitiers University Hospital, 2 rue de la Miletrie, 86000 Poitiers, France; 9 Diabetes Department, School of Cardiovascular and Metabolic Medicine, King’s College London, London, UK; 10 Institute of Diabetes, Endocrinology and Obesity, King’s Health Partners, London, UK

**Keywords:** Older adults, Cancer, Diabetes, Steroid-induced hyperglycaemia, Quality improvement, Multidisciplinary care

## Abstract

**Purpose:**

Older adults undergoing systemic anti-cancer therapy (SACT) with steroids are at high risk of developing steroid-induced hyperglycaemia (SIH). However, monitoring and management remain inconsistent. This quality improvement project (QIP) involved developing and evaluating the GOLD diabetes enhanced monitoring pathway, a multidisciplinary, age-adapted initiative for older patients at intermediate SIH risk.

**Methods:**

The pathway was implemented at Guy’s and St Thomas’ NHS Foundation Trust between February 2022 and June 2025. This included collaborative planning, structured risk stratification, diabetes education, home capillary blood glucose monitoring, telephone follow-up by geriatric clinical nurse specialists, medication and safety protocols. Iterative refinements were made through seven Plan-Do-Study-Act (PDSA) cycles. The primary outcome was the rate of SIH requiring diabetes medication intervention, whilst secondary outcomes included changes in SACT/steroid regimens, unplanned hospitalisations, and all-cause mortality.

**Results:**

Of the 126 patients (58% male; median age 74 years [IQR: 70–79]; 77% with pre-existing diabetes), diabetes medication intervention was provided in 32%, primarily with the initiation or escalation of gliclazide. Only three asymptomatic hypoglycaemic events occurred. Unplanned hospitalisations occurred in 28% of patients (*n = *35), with only two admissions related to diabetes. All-cause mortality (33%, *n = *41) was associated with metastatic disease, malnutrition, and social isolation, but not with glycaemic control or diabetes intervention.

**Conclusion:**

The GOLD pathway appears feasible, safe, and effective in managing SIH in older adults with cancer. Its success highlights the value of multidisciplinary collaboration and iterative refinement in addressing a critical gap in geriatric oncology care, as well as supporting structured diabetes monitoring in clinical practice. Larger multi-centre implementation effectiveness evaluation will enable sustainable, cost-effective practice across diverse settings.

**Supplementary Information:**

The online version contains supplementary material available at https://doi.org/10.1007/s41999-026-01585-w.

## Key summary points

**Aim:**

This study aimed to evaluate whether an age-adapted, multidisciplinary diabetes monitoring pathway could improve the management of steroid-induced hyperglycaemia in older adults undergoing systemic anti-cancer therapy.

**Findings:**

The GOLD pathway, implemented in 126 older patients, led to diabetes medication interventions in 34% of cases. Only three asymptomatic hypoglycaemic events occurred. Unplanned hospitalisations and all-cause mortality were not linked to glycaemic control. Iterative refinements and multidisciplinary collaboration were feasible and effective in this high-risk population.

**Message:**

Structured, age-adapted diabetes monitoring in geriatric oncology is both achievable and impactful, addressing a critical care gap for older adults receiving steroids during cancer treatment.

**Supplementary Information:**

The online version contains supplementary material available at https://doi.org/10.1007/s41999-026-01585-w.

## Introduction

Global ageing has led to an increasing incidence of cancer in people over the age of 65. This age group is characterised by an increased risk of multimorbidity and treatment-related complications [1, 2]. Diabetes stands out as a chronic condition with high prevalence amongst older adults with cancer, with up to 20% having a pre-existing diabetes diagnosis. It is also frequently exacerbated or triggered by cancer treatment, particularly with use of steroids [3–6]. Steroids are often an integral component of oncological treatment protocols or supportive care. However, steroid-induced hyperglycaemia poses a significant challenge in oncology, as it is associated with reduced overall survival, an increased risk of treatment toxicity and infection, higher rates of unplanned hospitalisation, and a greater likelihood of chemotherapy dose reduction or early discontinuation [7–11]. Systemic cancer treatments can also induce or exacerbate metabolic disorders, due to their impact on carbohydrate metabolism and pancreatic function [12]. Hyperglycaemia occurs in 10–30% of people undergoing chemotherapy [13]. Despite these risks, blood glucose monitoring during cancer treatment is inconsistent, exposing patients to potentially life-threatening complications such as the hyperosmolar hyperglycaemic state and diabetic ketoacidosis [14, 15]. Although collaborative management between oncology and diabetes teams exists, it is not systematically implemented in every cancer centre [12]. This gap in cancer care is particularly pronounced in older individuals, for whom the interactions between ageing, multimorbidity and complex treatment regimens demand a tailored, multidisciplinary approach [16].

In response to the unmet needs of this population, the Geriatric Oncology Liaison Development (GOLD) clinic at Guy’s and St Thomas’ NHS Foundation Trust has developed a structured, age-adapted enhanced monitoring pathway for diabetes, in collaboration with diabetes and oncology services. This pathway has been in place since 2022 [17]. The aim was to ensure a safe care plan for older patients considered to be at intermediate risk of treatment-induced hyperglycaemic events during systemic anti-cancer treatments (SACT) involving steroids. SACT include chemotherapy, immunotherapy, targeted therapy, and/or hormone therapy administrated to treat malignant diseases. Based on UK national guidelines, this multidisciplinary initiative addresses the specific vulnerabilities of older patients with cancer, by focussing on the proactive screening, monitoring, and individualised management of SACT and steroid-induced hyperglycaemia [12].

This manuscript describes the development, implementation, and evaluation of the GOLD diabetes enhanced monitoring pathway, using a quality improvement approach. Specifically, we aimed to: 1) describe the GOLD intervention, its improvement process, and the profiles of patients who were included in the enhanced diabetes monitoring pathway, and 2) evaluate the pathway’s feasibility and the safety, including the rate of steroid-induced hyperglycaemia requiring diabetes medication interventions and the incidence of adverse events.

## Methods

### Study design and context

This quality improvement project (QIP) was designed and reported in accordance with the Standards for QUality Improvement Reporting Excellence (SQUIRE 2.0) guidelines [18]. It was conducted within the GOLD clinic at Guy’s and St Thomas’ NHS Foundation Trust, a tertiary cancer centre in London, UK, working in collaboration with diabetes and oncology teams [17]. The GOLD clinic provides holistic multidisciplinary assessment and management for patients with solid or haematological malignancies who are aged ≥ 65 years, or ≥ 55 with significant complexity. The clinic’s objectives are to optimise patients’ health domains, including multimorbidity, physical and cognitive function, nutrition, mood, continence, general well-being and cancer care support. The geriatric team comprises four full-time nurses (two advanced nurse practitioners and two clinical nurse specialists, CNSs), three sessions of consultant geriatrician, one full-time registrar-grade doctor, one full-time physiotherapist, and one full-time occupational therapist.

The QIP addressed the local issue of the inconsistent monitoring and management of steroid-induced hyperglycaemia during SACT in older patients with cancer, despite national guidance and the well-known adverse outcome risks [12, 19]. The specific aims were to implement a structured, age-adapted diabetes enhanced monitoring pathway focussing on those aged ≥ 65 who are at intermediate risk and undergoing SACT with steroids, and to evaluate the feasibility, safety and clinical impact of the GOLD pathway (Supplementary Data 1) [20].

*Intervention*: development and implementation of the enhanced diabetes monitoring pathway.

### Planning the intervention

The intervention was developed in response to guidance on managing glycaemic control in patients with cancer, published by the UK Chemotherapy Board and the Joint British Diabetes Societies for Inpatient Care (JBDS-IP) in 2021 [12]. These national guidelines set out the principles for managing drug-induced hyperglycaemia in oncology to facilitate clinical practice, based on expert consensus. For this QIP, a multidisciplinary working group, including geriatricians, oncologists, diabetologists and CNSs, adapted the national recommendations to the local context, with a focus on considerations specific to geriatrics. During the pre-implementation phase, diabetes training was provided for the geriatric CNSs (12). Screening and decision-making algorithms were co-created, taking into account the feedback and experiences of each healthcare professional.

### Assessing the risk of steroid-induced hyperglycaemia

Baseline HbA1c and random glucose readings were used to evaluate hyperglycaemia risk, in line with national guidance reports stratified of management [12]. Low-risk patients were defined as having an HbA1c level of less than 48 mmol/mol (6.5%) and no history of diabetes mellitus. These patients were followed up by the oncology team and the general practitioner [21]. Conversely, high-risk patients requiring expert management were referred to the diabetes team. Initially, this group included patients treated with insulin, and those with an HbA1c level of ≥ 59 mmol/L (7.5%) or a capillary blood glucose (CBG) level of ≥ 15 mmol/L, as well as patients treated for a pancreatic cancer who often needed more tailored interventions [22]. Intermediate-risk patients, who did not meet the criteria for low or high risk, were referred to the GOLD team if they were 65 years old or older, and to the diabetes team if they were younger. Blood tests collected during cancer treatment in the oncology clinic were also considered as an entry point for enhanced monitoring.

### Providing patient education

In the GOLD diabetes enhanced monitoring pathway, the patient was referred by the oncology team, and provided with a CBG metre and education by either the GP or the GOLD team within a few days. Due to the very short time between referral and SACT initiation, this pathway must be responsive. The education phase was carried out by the geriatric CNS during a face-to-face meeting or a telephone call to assess the risk of steroid-induced hyperglycaemia and hypoglycaemia. They also collected medical data that could influence GOLD management, such as information on frailty and multimorbidity, renal and liver function, nutritional status and food intake. Patients were informed regarding risk of developing hyperglycaemia or hypoglycaemia and associated symptoms, need for CBG monitoring. They were also told how to promptly identify of diabetes emergencies (ketoacidosis, hyperglycaemic hyperosmolar syndrome, and hypoglycaemia). Glucose targets, practicalities and demonstrations of CBG monitor use were also covered during these meetings. The level of education provided was adapted to each patient’s competency, health literacy, and physical and cognitive abilities. When needed, education was simplified and involved caregivers. Communication aids were provided in accordance with the JBDS guidelines [12]. Standardised educational leaflets (developed by the GOLD team) were provided at the end of the discussion, either in person or via emails or telephone calls. Personalised tailored leaflets and handouts were created if necessary.

### Monitoring the risk of steroid-induced hyperglycaemia

During each SACT cycle, CBG levels were checked on the days of steroid administration and the following 2 days. CBG were checked before breakfast and evening meals, with the aim to capture peak of steroid-induced hyperglycaemia which occurs in the late afternoon or evening. Intervention and clinical management were based on a review of all glucose readings collected by the CNS via phone calls after each cycle of SACT. The recommended target glucose values were between 4.0 and 15.0 mmol/L. If abnormal readings were reported, the CNS asked for the time of the CBG reading (before or after eating) and the dose of medication taken. Hyperglycaemia was defined as at least two CBG readings higher than 15 mmol/L, and hypoglycaemia as one reading lower than four. Food intake and adverse symptoms were also assessed, with a particular focus on the onset of side effects of SACT, steroids, and hyperglycaemia. Symptoms of hypoglycaemia, whether during the day or at night, were monitored particularly closely. If necessary, interventions were provided in the form of advice and/or the start of medication, according to the algorithms. Dietetic recommendations were based on guidance regarding appropriate initial nutritional advice and referral to dietetic services. Advice also included the need to continue the CBG monitoring for a few extra days. Glucose testing was stopped if glucose readings were persistently below 12 mmol/L and SACT combined with steroids had been discontinued. Patients were then asked to arrange a follow-up appointment with their general practitioner.

### GOLD medication protocol for steroid-induced hyperglycaemia

Treatment regimens for patients with high glucose readings were outlined in decision-making algorithms. Therefore, diabetes medication should be initiated when blood glucose is ≥ 15 mmol/L on at least two occasions, whether morning or evening. A higher threshold of ≥ 15 mmol/L was chosen for our older population in our QIP, compared to the national guidance value of ≥ 12 mmol/L. This aligns with broader glycaemic targets for older people, helping to avoid hypoglycaemia and its consequences (12). In cases of steroid-induced hyperglycaemia involving at least two CBG readings higher than 15 mmol/L, the CNS discussed with the supervising doctor in geriatrics for the initiation of medication and dose escalation, if the protocol criteria were met and there were no contraindications (e.g. poor intake, renal impairment or risk of hypoglycaemia). In line with national guidance, gliclazide 40 mg once a day in the morning on steroid days would be the first-line intervention, unless there are any contraindications (12). If the target of below 15 mmol/L is not reached, the daily dose should be increased by 40 mg after each SACT cycle. Dose escalation depends on renal function, nutritional intake, frailty and risk of hypoglycaemia. The maximum total daily dose of gliclazide is 320 mg. This can be taken as a conventional dose of 160 mg twice daily, or 240 mg as a single morning dose to coincide with the steroids (with or without an evening dose up to 80 mg). If blood glucose levels remained high despite the maximum dose, the patient would be referred to the diabetes team. Patients taking gliclazide were counselled on the symptoms and management of potential hypoglycaemia. Patients with severe hepatic or renal impairment with eGFR cut-off of 30 mL/min were managed by the diabetes team as gliclazide should be avoided in these cases.

### Safety monitoring

Gliclazide initiation required a structured CNS-led approach to mitigate the risk of hypoglycaemia, particularly in patients with inconsistent oral intake and/or renal impairment. Patients were asked to report all readings particularly if out of target range. Telephone follow-ups immediately after each SACT cycle (from weekly to monthly) allowed the CNS to assess adherence and symptoms of hypoglycaemia. If CBG reading was below 6.0 mmol/L, gliclazide dose was reduced or stopped and hypoglycaemia education reinforced. The CNS would also reinforce education about emergency actions. If control was inadequate, dose escalation was considered with MDT oversight or diabetes team referral if gliclazide maximised.

### Implementing the QIP

The implementation of the GOLD diabetes enhanced monitoring pathway began in February 2022. Working group meetings were organised every 3 months from May 2022 to November 2023 to address HCP feedback with a proactive identification of barriers to implementation. From January 2023 onwards, fortnightly multidisciplinary meetings were held to review participants’ clinical cases, led by the geriatric CNS. Expert counselling was available to provide advice, medication or referrals. The pathway was iteratively refined using the PDSA (plan-do-study-act) cycles [23].

### Study of the intervention

#### Population and setting

The QIP included consecutive patients aged 65 years or older with histologically confirmed cancers, who were planned to undergo SACT with corticosteroids. Patients were classified by the oncology team as being at intermediate risk of SACT and steroid-induced hyperglycaemia, according to the criteria listed above. Patients were recruited from the South-East London Cancer Network between 1 February 2022 and 30 June 2025. Patients considered to be at high or low risk for SACT and steroid-induced hyperglycaemia were excluded from the study.

### Data collection

Data were collected prospectively from NHS system electronic medical records. Systematic extractions were performed, using pre-defined templates. A second blinded check was conducted retrospectively. The study incorporated data on oncology, geriatric and diabetes-related variables.

The oncological data included the primary cancer site, the presence of distant metastases and the cancer treatment strategy (SACT, including chemotherapy, immune therapy, and hormonal therapy; best supportive care). The protocol for the administration of steroid treatment was detailed, including the types of steroids, dose intensity, schedule and duration of treatment. The indications for steroid use were divided into four categories: use as part of the SACT regimen, use to treat immune therapy toxicity, use as part of a supportive treatment regimen during chemotherapy (e.g. as anti-emetic and anti-allergic medication) and other indications.

The geriatric data were extracted from the medical reports based on the GOLD comprehensive geriatric assessment (CGA) [24]. The following six CGA domains were considered in this study: social isolation; functional status with activities of daily living (ADL); risk of falls based on the timed get up and go test; history of recent falls (one or more falls in the six months prior to the assessment); cognitive status with history or medical diagnosis of dementia or cognitive impairment; mood with a medical diagnosis of depression and/or anxiety; and nutrition based on food intake reports, variation in weight and/or body mass index, and a medical diagnosis. Assessments of cognition and nutrition were clinical assessment based, rather than being based on the routine completion of validated tools such as the Mini Mental State Examination (MMSE), the Montreal Cognitive Assessment (MOCA) and the Mini Nutritional Assessment (MNA), unless there was a clinical indication to do so. Social isolation was considered in patients living alone without any caregiver, whether family or professional. The pre-treatment clinical frailty scale (CFS) was also documented, and categorised as follows: “Robust” (groups 1 and 2), “Living with pre-frailty” (groups 3 and 4) and “Living with frailty” (groups from 5 to 9) [25]. In accordance with guideline-based recommendations and patient preferences, personalised interventions are provided based on the identified geriatric impairments [26]. These interventions were categorised into four groups: physiotherapy, occupational therapy, dietary interventions and medication management. The following diabetes-related data were collected: history of pre-existing diabetes; existence of any vascular complications; anti-diabetes treatment, including diet, oral anti-diabetes drugs, and use of insulin. Vascular complications were categorised as cardiac complications (including a history of acute myocardial infarction, coronary artery disease or hospital admission for heart failure), peripheral vascular complications (including stroke, transient ischaemic attack, peripheral vascular disease, including previous revascularisation procedures and non-traumatic lower limb amputation), kidney complications (including diabetic kidney disease with a baseline creatinine clearance of less than 60 mL/min according to the Cockcroft and Gault formula, or renal replacement therapy), diabetic retinopathy and peripheral neuropathy [27]. The data from this study included HbA1c and random CBG levels from clinics, the number of SACT cycles under the GOLD monitoring, steroid regimens (in terms of medication type and duration), all fasting glucose readings (with morning and evening collection times), the need for advice and medication interventions (including type), and referral to the diabetes team for specialised clinical advice or monitoring (for patients classified as high risk afterwards). As previously stated, hyperglycaemia was defined as two or more readings of CBG higher than 15 mmol/L, and hypoglycaemia as one reading lower than four. Events related to glycaemia, both symptomatic and asymptomatic, were systematically recorded. The number and duration of phone calls made by the GOLD CNS were also documented. A standardised proforma was used for GP letters to ensure consistent information transfer for all the participants.

*The primary outcome was the*: rate of steroid-induced hyperglycaemia requiring diabetes medication intervention. The secondary outcomes included changes to the SACT protocol (e.g. completion, intensity and delay of treatment cycles) and steroid regimens whilst under GOLD supervision (e.g. dose adaptation). The onset of drug-induced side effects was also reported, as was cancer progression when the SACT and/or steroid regimens were impacted. All-cause mortality and unplanned hospitalisations were documented for each patient. Feedback was collected for the purpose of quality improvement. This included any barriers to implementing the GOLD pathway.

### Statistical analysis

The initial phase of this QIP involved the conduction of a descriptive analysis to report patients’ baseline characteristics. Continuous data were described using means ± standard deviations (SD) if they had a normal distribution, and median and interquartile (IQR, interquartile range) if not. Categorical variables were expressed as numbers and percentages (%). Patients’ characteristics were compared using a Student *t* test for continuous variables with a normal distribution, a Pearson’s Chi-square test for categorical variables, or a Wilcoxon signed-rank test for non-Gaussian distributions. As this QIP involves real-life processes, we have decided to include all participants in the analysis, even those with missing data.

The endpoints of interest were the occurrence of a medication intervention as the primary outcome, and overall survival and unplanned hospitalisations as the secondary outcomes. Survival analysis was conducted using Cox proportional hazards regression models. Comparisons of survival were expressed as hazard ratios (HRs) with 95% confidence intervals (95% CI). Statistically significant variables were defined as having a *p* value of less than 0.05, and all statistical tests were conducted using a two-tailed approach. The calculations were performed using the Statview^®^ software (SAS Institute, version 5.0).

### Ethical considerations

This QIP was registered and approved by the Guy’s and St Thomas’ NHS Foundation Trust audit and service evaluation governance team (project number: 18 525). Participants were asked to give their informed consent to take part in the monitoring pathway. Analysis of the anonymised data generated during this QIP was conducted in line with local information governance processes and guidance set out by the Health Research Authority. Further ethical approvals were not required.

## Results

### Initial steps of the interventions and their evolution over time

During the first 2 years, multidisciplinary working group meetings were held to develop operational and strategic links between the local oncology, diabetes and geriatrics services. The pathway was initially based on the JBDS-IP guidelines [12], and underwent refinement through seven Plan-Do-Study-Act (PDSA) cycles between 2022 and 2023 (Table 1). Each cycle incorporated feedback from healthcare professionals and addressed specific local barriers, such as the feasibility of glucose measurement, thresholds for intervention, and the decision-making process for initiating and titrating medication.

**Table 1:** Iterative refinement of the diabetes enhanced monitoring pathway using PDSA cycles

PDSA cycle	Plan	Do	Study	Act
Cycle 1 (2022)	Implement JBDS-IP guidance for glycaemic monitoring and risk stratification (5)	Applied HbA1c and venous glucose criteria for risk assessment	Found venous glucose measurement burdensome for oncologists	Adjusted to accept any venous/capillary/at-home glucose reading
Cycle 2 (2022)	Optimise detection of “at-risk” patients	Lowered glucose threshold for referral from 12 to 11 mmol/L	Improved detection of intermediate-risk patients	Maintained new threshold
Cycle 3 (2022)	Tailor pathway by age	Patients < 55 years to diabetes clinic; ≥ 55 years to GOLD clinic	Reduced diabetes team burden; improved geriatric management and expertise	Formalised age-based referral criteria
Cycle 4 (2022)	Anticipate high-risk hyperglycaemia	Lowered high-risk glucose threshold from 20.1 to 15 mmol/L	Increased early referrals to GOLD and diabetes team to avoid life-threatening complications	Maintained new threshold
Cycle 5 (2023)	Refine HbA1c referral criteria	Expanded intermediate-risk HbA1c range from 48–59 to 48–64 mmol/mmol	Reduced unnecessary referrals to diabetes team for older individuals; maintained safe management	Updated referral criteria
Cycle 6 (2023)	Broaden high-risk criteria	Added history of steroid hyperglycaemia as high-risk criteria	Improved identification of cases who may need complex interventions	Updated referral criteria
Cycle 7 (2023)	Empower geriatrics team for medication management	Allowed GOLD team to initiate then to escalate gliclazide up to 320 mg daily	Demonstrated safe and effective management without the need of diabetes team input	Formalised geriatrics-led medication management

**Fig. 1:**
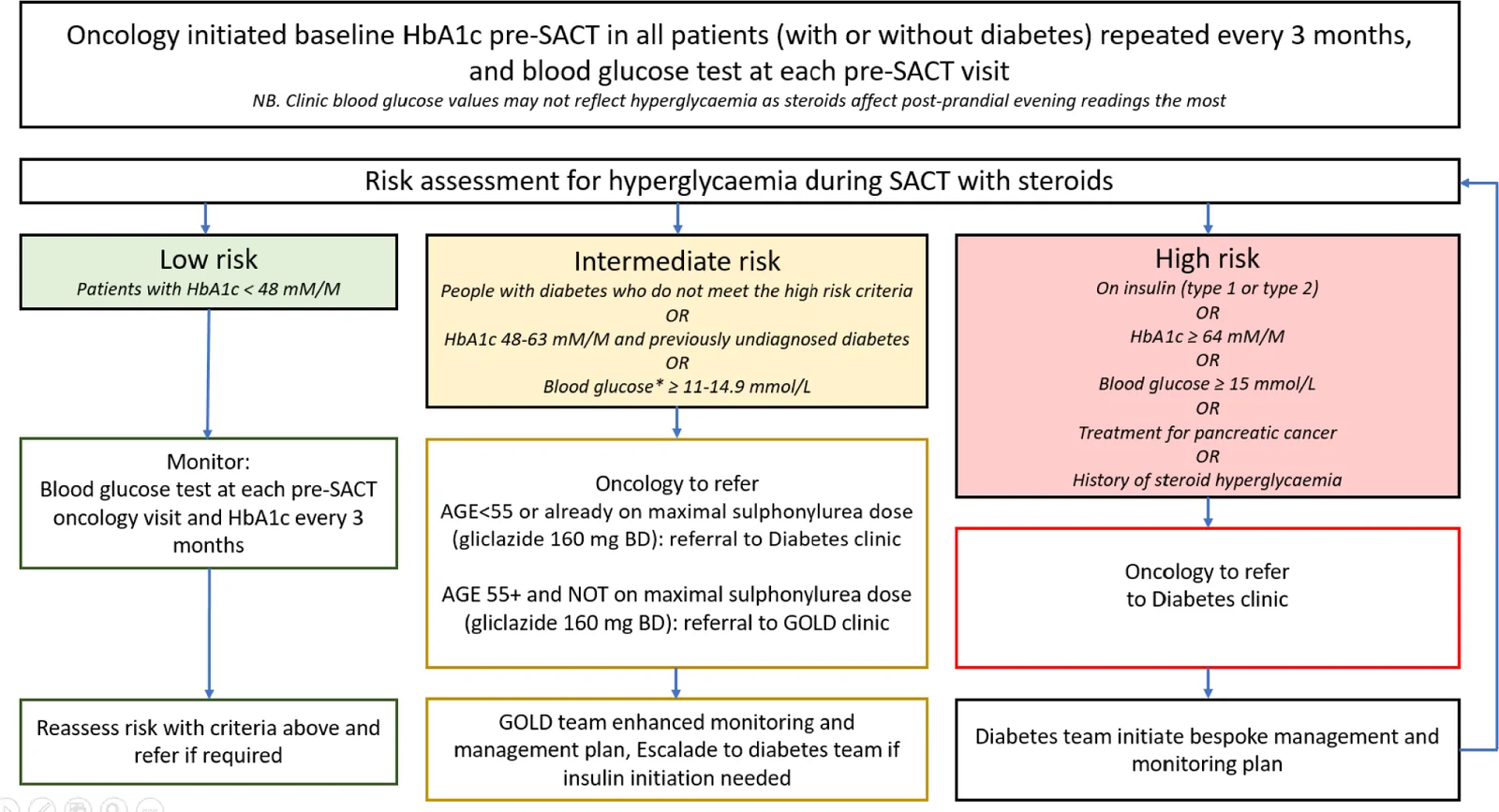
Risk assessment for steroid-induced hyperglycaemia and stratified diabetes pathway according to levels of risk (finalised pathway). *SACT* systemic anti-cancer therapy, *BD* twice a day *GOLD* geriatric oncology liaison development

Key QIP adaptations included three steps. First, an age-adapted referral was needed. Intermediate-risk patients aged 65 years or over were managed by the GOLD team, whilst younger patients were referred to the diabetes clinic, in line with local practices for geriatric oncology referrals. Second, the definition of risk stratification was defined as follows (Fig. 1). Patients were classified as high risk if they met one or more of the following criteria: insulin use, HbA1c ≥ 64 mmol/mol (8%), glucose ≥ 15 mmol/L or pancreatic cancer. Patients with a past history of steroid-induced hyperglycaemia were also considered to be at high risk, due to CNS feedback and frequent referrals for specialist diabetes advice. Patients were considered to beat intermediate risk if they had an HbA1c level of 48–63 mmol/mol (6.5–7.9%), or a capillary glucose level of 11–14.9 mmol/L, or if they had diabetes and were on oral glucose-lowering therapies but did not meet the high-risk criteria. In six of the seven PDSA cycles, the risk assessment tool was modified (Table 1). Specifically, the glucose threshold was adjusted from 12–20 to 11–15 mmol/L, and the HbA1c range was expanded from 48–59 to 48–64 mmol/mol to refine the identification of the intermediate-risk group for GOLD referral. Finally, the monitoring and management protocols were validated (Supplementary Data 1).

### Patient population and baseline characteristics

**Fig. 2:**
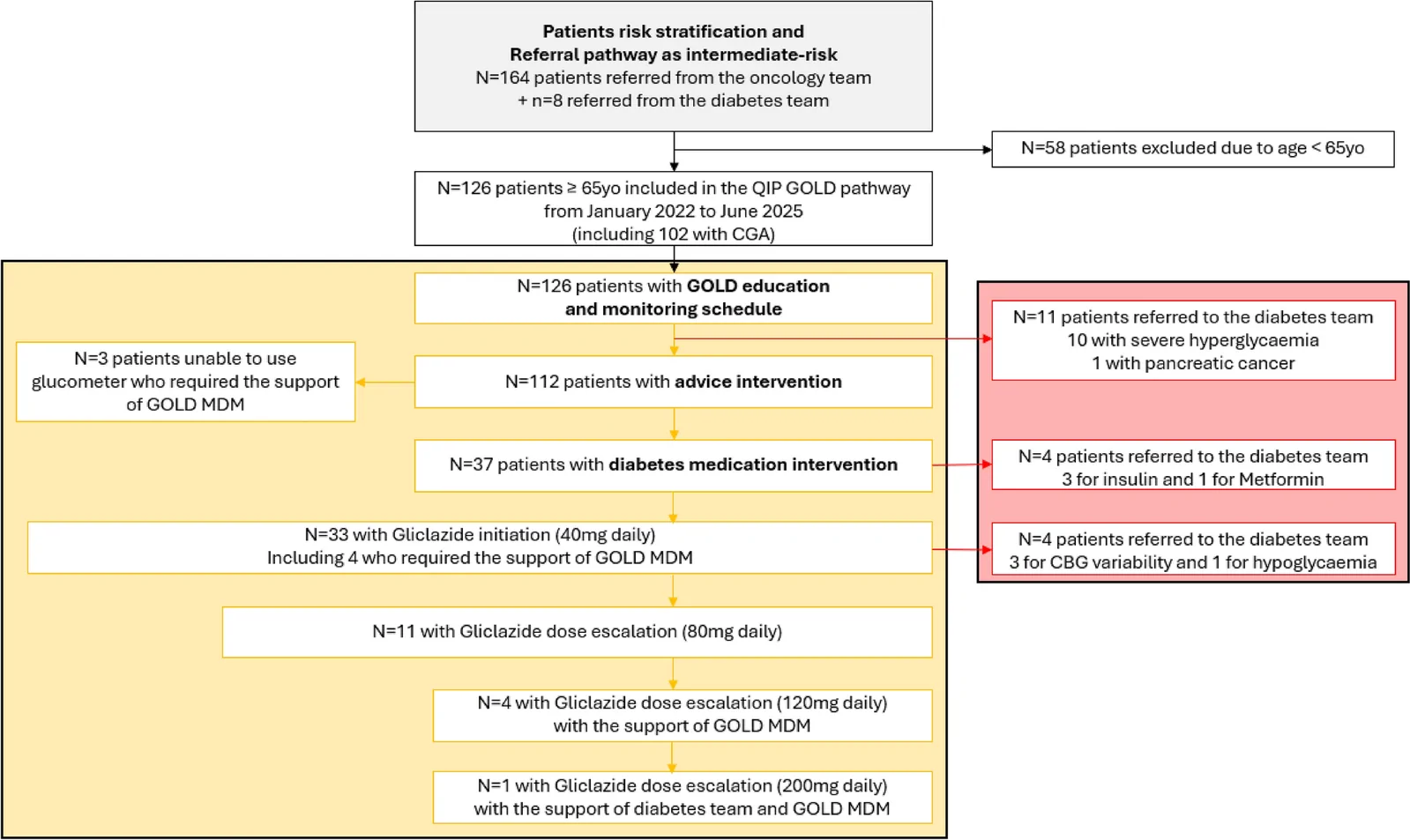
Flow diagram of the GOLD pathway. *yo* years old, *QIP* quality improvement project, *GOLD* geriatric oncology liaison development, *CGA* comprehensive geriatric assessment, *MDM* multidisciplinary meeting

In total, 126 patients (median age 74 years [70–79]; 58% male) were enrolled in the monitoring pathway (Table 2, Fig. 2). The most common primary tumour sites were oesophagus (*n = *18, 14%), colon (*n = *15, 12%) and breast (*n = *11, 9%). Of these patients, 44% presented with metastatic disease. In this study, 94% of participants received chemotherapy as their primary systemic anti-cancer therapy. The most common steroid regimen involved dexamethasone, with a median daily dose of 8 mg (ranging from 4 to 20 mg, equivalent to 27 mg of prednisone), for a short period, as it was administered for the first 4 days of each SACT cycle. It was primarily used as supportive treatment during chemotherapy for its anti-emetic and anti-allergic properties (70%). Prednisolone was prescribed less frequently, either as part of SACT regimens (e.g. 100 mg per day for five days in R-CHOP for lymphoma) or for palliative symptom control (0.5 to 1 mg/kg/day for cerebral oedema). An history of diabetes was noted in 77% (*n = *97), 54% were treated with oral antidiabetic monotherapy, 21% with a combination of oral medications, 3% with insulin (these patients were recruited in 2022, before the intermediate risk criteria changed) and 23% with dietary interventions alone. Of this group, 37% reported vascular complications, most commonly cardiac (23%) and renal (13%). Amongst those with pre-existing diabetes, CGA data were available for 102 patients (81%), revealing a median CFS score of 3 [IQR 3–4]. Data from CGAs were unavailable for 19% of patients, primarily due to individuals being excluded during triage on the basis that they were unlikely to benefit, time limitations or patient refusal. Malnutrition, as determined by medical judgement, was identified in 46% of the cohort, whilst social isolation, functional impairment and recent falls were reported in 6%, 12% and 14% of patients, respectively. Personalised geriatric interventions were implemented for all patients diagnosed with impaired geriatric domains (*n = *82, 81%).

**Table 2:** Baseline characteristics of the population (*n = *126)

Patient characteristics	
Demographics	
Age in years, median [Q1-Q3]	74.0 [70.0–79.0]
Gender male, *n* (%)	73 (58%)
Ethnicity, *n* (%)	
White	49 (39%)
Black or Black British	36 (29%)
Asian or Asian British	5 (4%)
Mixed	1 (1%)
Others	3 (2%)
Not stated/no data available	32 (25%)
Oncology characteristics	
Main tumour sites, *n* (%)	
Oesophagus	18 (14%)
Colon	15 (12%)
Breast	11 (9%)
Myeloma	11 (9%)
Endometrium	10 (8%)
Lung	10 (8%)
Stomach	9 (7%)
Ovary	9 (7%)
Prostate	8 (6%)
Others*	25 (20%)
Metastatic status, *n* (%)	53 (44%)
Cancer treatment, *n* (%)	
Chemotherapy	112 (89%)
Combination therapy	11 (9%)
Immune monotherapy	2 (1%)
Hormone therapy	1 (1%)
Diabetes characteristics	
Pre-existing diabetes, *n* (%)	97 (77%)
Diabetes with any vascular complications, *n* (%)	36 (37%)
Heart complication	22 (23%)
Renal complication	13 (13%)
Peripheral vascular complication	5 (5%)
Diabetic retinopathy	8 (8%)
Diabetic neuropathy	6 (6%)
Initial diabetes treatment, *n* (%)	
Oral monotherapy	52 (54%)
Diet	22 (23%)
Combination oral therapy	20 (21%)
Insulin therapy	3 (3%)
HbA1c at GOLD clinic (*n = *125) in mmol/L	51 [48–56]
HbA1c at GOLD clinic (*n = *125) in %	6.8 [6.5–7.3]
Random glucose in GOLD clinic (*n = *109) in mmol/L	7.90 [6.40–9.80]
Geriatric characteristics	
GOLD comprehensive geriatric assessment, *n* (%)	102 (81%)
CFS (*n = *127), median [Q1-Q3]	3 [3, 4]
CFS = 2 (considered as fit)	16 (16%)
CFS = 3 (managing well)	39 (40%)
CFS = 4 (living with very mild frailty)	25 (26%)
CFS = 5 (living with mild frailty)	11 (11%)
CFS = 6 (living with moderate frailty)	4 (4%)
CFS = 7 (living with severe frailty)	2 (2%)
Social isolation, *n* (%)	6 (6%)
Dependency in ADL, *n* (%)	12 (12%)
Timed get up and Go (*n = *75), median in seconds	12.9 [10.7–18.6]
History of falls (≥ one in the previous 6 months), *n* (%)	14 (14%)
Cognitive impairment, *n* (%)	21 (21%)
Low mood (anxiety and/or depression), *n* (%)	18 (18%)
Malnutrition, *n* (%)	46 (46%)
GOLD interventions other than DP, *n* (%)	82 (81%)
Physiotherapy	31 (31%)
Occupational therapy	18 (18%)
Drug management	53 (52%)
Diet management	55 (54%)

^*^Other tumour sites: cholangiocarcinoma, *n = *5; lymphoma, *n = *5; bladder, *n = *3; rectum, *n = *3; head and neck, *n = *1; anal, *n = *1; liver, *n = *1; pancreas, *n = *1; ampullary, *n = *1; cervix, *n = *1; MGUS, *n = *1; skin, *n = *1; unknown, *n = *1

*ADL* activities of daily livings, *CFS* clinical frailty scale, *DP* diabetes pathway, *GOLD* geriatric oncology liaison development, *MGUS* monoclonal gammopathy of undetermined significance, *SD* standard deviation

### Implementation and outcomes of the quality improvement intervention

#### Process measures

The GOLD pathway was implemented as a structured, iterative quality improvement project, with process measures collected to assess its feasibility (Table 1). The number of patients under GOLD monitoring increased over time, from 25 individuals in 2022 to 22 from January to June 2025 (compared to 24 in 2023, and 55 in 2024). Patients were monitored for a median of three SACT cycles, with a median of four telephone follow-up calls per patient (ranging from one to 16 calls, Table 3). The estimated length of each phone call was estimated between 5 and 24 min.

**Table 3:** Characteristics of the GOLD diabetes enhanced monitoring pathway (*n = *126)

Cancer treatment cycles under DP supervision, median [Q1-Q3]	3 [1–5]
	range 1–15
Number of phone calls made (*n = *109), median [Q1-Q3]	4 [2–7]
	range 0–16
CBG readings in mmol/L, median [Q1-Q3]	8.52 [7.70–10.00]
CBG AM readings in mmol/L, median [Q1-Q3]	7.45 [6.71–8.49]
CBG PM readings in mmol/L, median [Q1-Q3]	10.00 [8.48–11.73]
Highest CBG reading in mmol/L, median [Q1-Q3]	14.85 [12.25–19.12]
Referred to diabetes team, n (%)	15 (12%)
Referred from diabetes team, n (%)	8 (6%)
Supported by diabetes team with clinical advice, n (%)	17 (13%)
Diabetes advice provided to the patient, *n* (%)	112 (89%)
New diabetes medication intervention needed, *n* (%)	37 (32%)

*AM* morning time, *CBG* capillary blood glucose, *DP* diabetes pathway, *GOLD* geriatric oncology liaison development, *PM* evening time

Three patients (2%) were unable to use the CBG device and were therefore not able to report any readings. For these patients, glucose values were recorded at each hospital visit, and HbA1c levels were monitored every 3 months in collaboration with the oncology team and/or the general practitioner. Telephone GOLD follow-ups were organised, to assess symptoms that might suggest either hyperglycaemia or hypoglycaemia.

Amongst the remaining participants with available CBG values, the median fasting blood glucose level was 8.52 mmol/L (IQR 7.70–10.00). Higher values were observed in the evening. Clinical advice on diabetes management was provided to 89% of patients (*n = *112, Table 3). The need for medication intervention was identified in 32% of patients (*n = *37), but did not differ according to frailty levels (*p = *0.75, Supplementary Data 2). Of these patients, gliclazide was initiated or escalated in 34 cases (92%). Drug-induced hypoglycaemia occurred in three patients (with glucose readings of 2.0, 3.5 and 3.6mmol/L) following the introduction of oral antidiabetic medication. These acute events were asymptomatic. Specialised clinical advice was required for four patients (8%): insulin therapy was needed for three patients, and the metformin dose was increased for one patient.

Multidisciplinary collaboration was a key component of this pathway. Eight patients (6%) were initially referred to the diabetes team before transitioning to the GOLD pathway, whilst fifteen patients (12%) required escalation from the GOLD pathway to the diabetes team due to severe hyperglycaemia. Secondary referrals to the diabetes team were more common at the beginning of the QIP period (36% of patients enrolled in 2022, compared to 10% in 2024, and 5% in 2025, *p = *0.03). Fortnightly multidisciplinary team meetings validated all the patients’ pathways and facilitated the collaborative decision-making process in 17 cases (13%).

### Primary and secondary outcomes

#### Primary outcome: diabetes medication intervention

The primary outcome, the rate of steroid-induced hyperglycaemia requiring diabetes medication intervention, was observed in 32% of patients. Female participants and patients with pre-existing diabetes were significantly more likely to require intervention (respectively, 62% versus 37%, *p = *0.01, and 89% versus 70%, *p = *0.02, Table 4). However, no association was found between hyperglycaemia and any geriatric domains, or oncological characteristics. A sensitivity analysis excluding patients with missing CGA data was performed, which did not alter the primary findings (Supplementary Data 3).

**Table 4:** Association amongst clinical and biological characteristics and steroid-induced hyperglycaemia in the GOLD patients

Variables	Steroid-induced hyperglycaemia (*n = *37)	No steroid-induced hyperglycaemia (*n = *79)	*p* value
Age in years, median [Q1-Q3]	72.0 [69.0–79.0]	76.0 [70.5–79.5]	0.14
Sex female, *n* (%)	23 (62)	29 (37)	0.01
Metastatic status, *n* (%)	15 (41)	31 (42)	0.89
Pre-existing diabetes, *n* (%)	33 (89)	55 (70)	0.02
CFS, median [Q1-Q3]	3.00 [3.00–4.00]	3.00 [3.00–4.00]	0.99
Social isolation, n (%), *n = *102	1 (3)	3 (5)	0.99
Dependency in ADL, *n* (%), *n = *102	3 (10)	9 (15)	0.74
Timed get up and go test (sec, median [Q1-Q3]), *n = *102	12.6 [10.3–16.3]	13.9 [11.2–19.9]	0.26
History of falls, *n* (%), *n = *102	5 (17)	8 (13)	0.75
Cognitive impairment, *n* (%), *n = *102	2 (7)	16 (26)	0.05
Low mood, *n* (%), *n = *102	5 (17)	12 (20)	0.78
Malnutrition, *n* (%), *n = *102	13 (45)	28 (46)	0.92
Other holistic interventions (%)	24 (80)	50 (82)	0.82
HbA1c at GOLD clinic (mmol/L, median [Q1-Q3])	53 [48–58]	50 [46–55]	0.12
HbA1c at GOLD clinic (%, median [Q1-Q3])	7.0 [6.5—7.5]	6.7 [6.4–7.7]	0.12
Random CBG in GOLD clinic (mmol/L, median [Q1-Q3])	8.55 [6.82–10.88]	7.70 [6.50–9.40]	0.08
All CBG readings (mmol/L, mean ± SD)	10.82 ± 2.03	8.13 ± 1.18	< 0.001
Glucose AM readings (mmol/L, mean ± SD)	9.21 ± 2.22	7.25 ± 1.18	< 0.001
Glucose PM readings (mmol/L, mean ± SD)	12.56 ± 2.54	9.14 ± 1.52	< 0.001
Highest glucose reading (mmol/L, median [Q1-Q3])	20.30 [18.70–22.20]	12.80 [11.70–15.10]	< 0.001
Number of cancer treatment cycles (median, [Q1-Q3])	4 [2;6]	3 [1;5]	0.19
Any change in cancer treatment plan during CBG monitoring, *n* (%)	17 (47)	36 (48)	0.94
Any change in steroid regimens during CBG monitoring, *n* (%)	16 (43)	20 (25)	< 0.001

*ADL* activities in daily living, *AM* morning time, *CBG* capillary blood glucose, *CFS* clinical frailty scale, *GOLD* geriatric oncology liaison development, *PM* evening time, *SD* standard deviation

#### Secondary outcomes: treatment adaptations in SACT and steroids, overall mortality and hospital readmissions

During the QIP period, 47% of patients (*n = *53) required a modification to their SACT plan. The study revealed a significant reduction in SACT doses for 42 patients. Notably, treatment was discontinued for 35 patients, primarily due to SACT side effects (51%) and cancer progression (25%). No SACT intensification was reported. The need for gliclazide in patients with a higher rate of raised CBG was not associated with the SACT adaptation (*p = *0.94, Table 4).

All changes to steroid regimens were decided and validated by the oncologist in 30 patients (24%), all of which consisted of a steroid reduction. The most common adaptation was a reduction in the daily steroid dose (73%), followed by the shorter treatment duration per SACT cycle (19%) and early discontinuation of steroids (8%). This dose adjustment was decided in response to hyperglycaemia in 80% of cases. It was driven by the need for intervention with diabetes medication (Table 4). Following the reduction in steroid treatment, four patients experienced symptoms of SACT-induced toxicity, such as fatigue, nausea and loss of appetite. This resulted in the early discontinuation of the anti-cancer protocol for two individuals and a reduction in dose intensity for the other two. There was no difference in the initial steroid dose intensity between patients requiring diabetes medication intervention and those who did not. Significant associations have been identified between steroid reduction and changes in SACT regimens (p < 0.001).

During a median follow-up period of 300 days (IQR: 178–566), 41 deaths (32%) were recorded. All-cause mortality was significantly associated with metastatic status (HR = 2.43, 95%CI = 1.23–4.81, *p = *0.01), social isolation (HR = 3.34, 95%CI = 1.15–9.68, *p = *0.03) and malnutrition (HR = 3.00, 95% CI = 1.45–6.20, p < 0.001, Supplementary Data 4). No significant association was found between mortality and the need for intervention with diabetes medication (*p = *0.35), pre-existing diabetes (*p = *0.17) or any glycaemic readings (*p = *0.32).

Within 1 year of CGA, 35 patients (28%) experienced unscheduled hospital admissions, with only 2 hospitalisations related to hyperglycaemia (glucose readings of 26 and 32 mmol/L). There were no reports of diabetic ketoacidosis or hyperglycaemic hyperosmolar syndrome. No admissions were related to hypoglycaemia. There was no significant difference in readmission rates observed between patients who received diabetes medication and those who did not (35% and 24% respectively, *p = *0.08).

### Iterative learning and barriers to implementation

The pathway was refined through seven Plan-Do-Study-Act (PDSA) cycles (Table 1), which addressed issues relating to feasibility, risk stratification, education, monitoring and management algorithms, including medication and safety protocols. Key barriers included patient difficulty with glucometer use (*n = *6), highlighting the need for enhanced education and support (*n = *77, 47%), and logistical challenges in equipment provision (*n = *52 via GOLD, *n = *42 via the general practitioner, *n = *7 via the diabetes team).

Facilitators of successful implementation included: multidisciplinary collaboration between geriatrics, oncology, diabetes and primary care teams; fortnightly team meetings to review complex clinical cases (e.g. patients with severe hyperglycaemia, or difficulty with glucometer use) and refine the personalised pathway; and geriatrics team increasing their autonomy in managing intermediate-risk patients, thereby reducing the burden on diabetes specialists.

## Discussion

This quality improvement project (QIP) demonstrates the feasibility and the clinical value of a structured, multidisciplinary diabetes enhanced monitoring pathway for older patients with cancer undergoing SACT combined with steroids. The GOLD pathway was developed using continuous quality improvement tools and iterative PDSA cycles. This new pathway, which has been tailored to the needs of older intermediate-risk patients, has been implemented on a sustained basis. It offers a practical model for integrating geriatric, oncology and diabetes care in a routine setting.

Our results confirm that the GOLD pathway was successfully implemented. The number of monitored patients increased over time, whilst the rate of adverse effects and unplanned hospitalisations for diabetes-related events remained low. These outcomes were observed even in patients who experienced drug-induced hyperglycaemia and required a diabetes medication intervention. Importantly, only three cases of asymptomatic hypoglycaemia were observed, all of which were managed without clinical consequences. This safety profile seems to be interesting given the increased risk of hypoglycaemia in patients with cancer, which can be attributed to poor oral intake and digestive symptoms, such as nausea and vomiting that are caused by the cancer itself or its treatment. However, the low rate of hypoglycaemia (three asymptomatic events) suggests that the pathway was safe in this cohort, though under-ascertainment of nocturnal or asymptomatic events cannot be excluded. It may also be indicative of permissive glycaemic targets or a relatively low frailty burden. Using continuous glycaemic monitoring would allow to detect asymptomatic glycaemic variations, including at night, as our QIP study was not designed to monitor glycaemic values during these hours. Older age has been identified as a risk factor for both hyperglycaemia and hypoglycaemia [5, 28]. The low rate of diabetes-related hospitalisations suggests that proactive monitoring, patient education and timely interventions could help to prevent severe hyperglycaemic events. These results emphasise the importance of monitoring processes and outcome measures when implementing a complex intervention. Furthermore, our findings highlight the need for multidisciplinary collaboration when addressing this challenging clinical area. The person-centred nature of the pathway, engaging with oncologists, diabetologists, geriatricians, general practitioners and patients, was essential in achieving these outcomes [29, 30]. This collaborative approach is particularly important for older people with multimorbidity who have been diagnosed with cancer, as they are at an increased risk of drug-induced hyperglycaemia and diabetes. To improve the care and quality of life in patients with cancer, it is important to recognise, manage and initiate antidiabetic therapy at an early stage in cases of anti-cancer drug-induced hyperglycaemia. This can be achieved through effective teamwork involving communication, coordination and cooperation between professionals, Other healthcare professionals and caregivers should receive ongoing training and education. This ensures that they can recognise and treat clinical cases before they escalate to acute, life-threatening conditions [31]. Thanks to this QIP, the GOLD team has developed valuable expertise in both diabetology and oncology. The team has become increasingly autonomous, in particular the nursing staff. They have gained experience in managing complex situations and administering medication, whilst retaining the option of referring cases or seeking advice from the diabetes team if specialist input is required.

The medication protocol relies on gliclazide, as a reasonable choice for managing steroid-induced hyperglycaemia in older patients, in line with the JBDS-IP/UK Chemotherapy Board’s recommendations [12]. This sulphonylurea was chosen because it can respond to the speed of onset and extent of steroid-induced hyperglycaemia. Steroids increase insulin resistance and impair beta-cell function, resulting in hyperglycaemia. Gliclazide directly stimulates insulin secretion, counteracting the steroid-induced suppression of beta-cell activity. As an inexpensive oral agent, gliclazide is practical for outpatient management, and avoids the potential burden of injectable therapies, such as insulin. Whilst nutritional intake can be variable, steroid-induced hyperglycaemia is generally independent of this. Patients systematically receive education and advice about the risks of hypoglycaemia. Alternative treatments may include an insulin-based approach (e.g. an intermediate acting insulin such as Humulin I, taken once or twice a day to match the hyperglycaemic profile of the steroids). This may be preferred in cases of severe hyperglycaemia where sulphonylureas are ineffective or contraindicated. Adjustment and escalation of insulin regimens under the guidance of a diabetes team takes into consideration other factors such as intermittent or meaning steroid dosing, appetite and weight changes, feeds or supplements. Other glucose-lowering therapies, such as metformin or DPP4 inhibitors, are generally less effective and have a slower onset of action in cases of steroid-induced hyperglycaemia [32]. Whilst they may be considered on an individual basis, particularly in patients with suboptimal baseline glycaemic control, sulfonylureas and insulin remain the cornerstone of management. Care must also be taken regarding side effects of SACT that overlap with those of potential oral agents, such as diarrhoea, and the risk of lactic acidosis in patients with renal impairment taking metformin.

Gliclazide was effective for most patients at intermediate risk with only three progressing to insulin treatment. Initially managing with oral agents before progressing to insulin if needed reduces the unnecessary burden of insulin injections. Surprisingly, there is no randomised trial evidence to suggest that tight glycaemic control improves outcomes in oncology. Furthermore, research on the collaborative management of glycaemic balance in oncology settings is limited [33, 34]. This is an area that requires urgent research [35]. The emerging field of diabetology in oncology aligns with the philosophy of geriatric oncology, focussing on a holistic, person-centred approach involving monitoring, implementing individualised corrective interventions and conducting close follow-up. This approach aims to significantly enhance patients’ quality of life and prevent drug side effects [36–38]. Further prospective studies are needed to validate the care model in diabeto-oncology in other settings, and could draw inspiration from care plans in geriatric oncology. Model-based strategies (e.g. the screen-and-refer model, the shared-care model, the multidisciplinary consultative model, the diabetology-embedded consultative model, the diabeto-oncologist as a primary provider or the patient self-management) present an opportunity to validate an efficient collaborative management approach [39].

Several limitations should be kept in mind. First, this QIP featured a single-centre design, and included patients who were mostly pre-frail. This may affect the generalisability of our findings to older adults who live with frailty. Second, the geriatric assessment was not systematic, and some domains (such as cognition and malnutrition) were not evaluating using validated tools, which may affect the reproducibility of the results. Third, whilst the pathway was associated with low rates of severe hyperglycaemia and hypoglycaemia, the absence of a comparator group limits our ability to attribute these outcomes directly to the intervention. Future studies should include pre–post comparisons or controlled designs to assess effectiveness. And finally, integrating patient-reported outcomes, cancer-related outcomes and a cost-effectiveness analysis into the identification of adverse events could ensure the robustness of our QIP strategy.

Patient engagement and experience are central to the success of any quality improvement initiative. Whilst most patients successfully participated in the pathway, a small subset faced challenges, primarily related to the use of glucometer. This highlights the importance of providing personalised patient education and support, particularly for older adults who may struggle with health or technology literacy [31, 40]. Future PDSA cycles aimed at improving this pathway should incorporate patient feedback to enhance usability and accessibility, thereby ensuring that everyone can benefit from enhanced monitoring. It should be noted that MDT working could improve patient experience by reducing the number of appointments for patients who already have multiple cancer appointments.

The GOLD pathway aligns with national and international recommendations for an integrated, multidisciplinary care for older adults with complex health needs [10, 41–43]. Based on a pragmatic approach, the absence of a validated tool to screen risk levels may limit the generalisability of our pathway. In future iterations, validation of reproducible risk-based stratification could be incorporated into the QIP process.

In our QIP, 81% of the patients underwent CGA; personalised corrective interventions were systematically implemented and monitored when an altered geriatric domain was diagnosed. The GOLD diabetes enhanced monitoring pathway may have included time dedicated to the education and monitoring of geriatric syndromes, in addition to diabetes care. This integrative management approach may explain the low rate of adverse outcomes. Our findings support the adoption of structured diabetes monitoring pathways in geriatric oncology services. Policymakers and healthcare leaders should consider incorporating such pathways into standard cancer care protocols, and allocating the necessary resources and training to support their implementation [44].

In our pathway, the time investment required from CNSs is a notable resource consideration. Whilst the pathway’s effectiveness justifies this allocation, integrating digital health tools, such as remote glucose monitoring and digital devices, could enhance efficiency, particularly in settings with limited resources [45].

Furthermore, the success of this QIP highlights the importance of quality improvement methodologies in addressing care gaps for older adults. Although national guidelines, such as those from the JBDS-IP, provide a valuable framework, local adaptation and iterative refinement, as demonstrated here, are essential for optimising outcomes in diverse clinical settings [12]. The sustainability of our enhanced pathway needs to be validated over time, as it has only been in place for 3 years [46].

Whilst this QIP did not formally assess cost-effectiveness, the potential economic benefits of the GOLD pathway warrant further investigation. Proactive diabetes management could reduce costly complications, such as unplanned hospitalisations and treatment interruptions, which are associated with significant healthcare expenditure [47]. Future studies should evaluate the cost-effectiveness of the pathway, by comparing the initial investment in monitoring and education with the savings from preventing adverse events. Such data would strengthen the case for the wider adoption and funding of similar initiatives.

The GOLD pathway provides a robust model for personalised, tailored interventions in older intermediate-risk patients with cancer and diabetes. To build on these findings, future research should focus on the following: conducting prospective comparative studies to confirm the pathway’s efficacy in preventing adverse outcomes and improving quality of life. This would help to ensure the reproducibility of this pathway, enabling its implementation in other settings through multi-centre studies. Integrating digital health solutions would explore streamline monitoring and reduce staff workload. Assessment of patient-reported outcomes should include longitudinal evaluations of quality of life, treatment satisfaction, and personal experiences with the GOLD pathway. Finally, the evaluation of its cost-effectiveness will inform policy and funding decisions. The ultimate goal would be to expand the diabetes enhanced monitoring pathway to other healthcare settings. This would allow to assess its generalisability and adaptability for implementation.

## Conclusion

Based on a QIP pragmatic design, the GOLD enhanced monitoring pathway seems to be a feasible patient-centred approach to managing diabetes in older patients with cancer who are undergoing SACT with steroids. However, our findings should be validated in future randomised trials to confirm its effectiveness, safety and clinical impact. Leveraging multidisciplinary collaboration and iterative quality improvement, this model addresses a key issue in the field of geriatric oncology. Further work should focus on validating its benefits, exploring its cost-effectiveness, and incorporating patient perspectives to refine and expand its impact further.

## Supplementary Information

Below is the link to the electronic supplementary material.

Supplementary file1 (DOCX 147 KB)

## Data Availability

Data sets are available on reasonable request.
