# Supplementary material for: Improving diabetes management in older adults with cancer: a quality improvement project to enhance monitoring during steroid-based systemic anti-cancer therapy
Source: Eur Geriatr Med. 2026 Sep 3;17(5):3037–51. doi: 10.1007/s41999-026-01585-w (PMC13754967; doi:10.1007/s41999-026-01585-w)
Supplement: Supplementary file 1 [file 41999_2026_1585_MOESM1_ESM.docx]

**Improving Diabetes Management in Older Adults with Cancer: A Quality Improvement Project to Enhance Monitoring During Steroid-Based Systemic Anti- Cancer Therapy**

Journal of European Geriatric Medicine

**Author names:** Evelyne Liuu; Rose Evans; Gitanjali Amaratunga; Sarah Compton; Alexandros Georgiou; Tania Kalsi; Yuk-Fun Liu; James Maguire; Clare Nottage; Hannah L. Rush; Carly Welch

**Affiliations and e-mail address of the corresponding author:**

^a^ Department of Ageing and Health, 9th Floor North Wing, St Thomas’ Hospital, Westminster Bridge, London, SE1 7EH, United Kingdom

^b^ Department of Twin Research & Genetic Epidemiology, 3rd Floor D Block South Wing, St Thomas’ Campus, King’s College London, London, SE1 7EH, United Kingdom

^c^ University of Poitiers, INSERM 1402, CHU Poitiers, Centre d’Investigation Clinique, Laboratoire MOVE, department of geriatrics, 2 rue de la Miletrie, 86000 Poitiers, France

[evelyne.liuu@univ-poitiers.fr](mailto:evelyne.liuu@univ-poitiers.fr)

**Supplementary data 1**: GOLD enhanced monitoring pathway

Abbreviations: CBG, capillary blood glucose in mmol/L; GOLD, geriatric oncology liaison development; CNS, clinical nurse specialist; GP, general practitioner; A&E, accident and emergency department; SACT, systemic anticancer therapy; OD, once a day; AM, in the morning.

# **Supplementary data 2:** Association among clinical and biological characteristics and frailty level (according to CFS categories) in the GOLD patients

| **Variables** | **FIT**  **CFS 1-2 (n=16)** | **PREFRAILTY**  **CFS 3-4 (n=64)** | **FRAILTY**  **CFS 5-9 (n=17)** | **P-value** |
| --- | --- | --- | --- | --- |
| CFS, median [Q1;Q3] | 2.00 [2.00;2.00] | 3.00 [3.00;4.00] | 5.00 [5.00;6.00] | <0.001 |
| Age in years, median [Q1;Q3] | 74.0 [70.8;79.2] | 74.0 [69.8;80.0] | 76.0 [70.0;78.0] | 0.97 |
| Sex female, n (%) | 4 (25%) | 26 (41%) | 9 (53%) | 0.26 |
| Metastatic status, n (%) | 6 (38%) | 27 (45%) | 7 (41%) | 0.85 |
| Pre-existing diabetes, n (%) | 10 (62%) | 53 (83%) | 14 (82%) | 0.19 |
|  |  |  |  |  |
| Social isolation, n (%) | 0 (0%) | 4 (6%) | 2 (12%) | 0.48 |
| **Dependency in ADL, n (%)** | **0 (0%)** | **1 (2%)** | **10 (59%)** | **<0.001** |
| **Timed get up and go test (sec), median [Q1;Q3]** | **10.2 [9.0;10.7]** | **13.3 [11.5;17.3]** | **20.1 [13.4;29.1]** | **<0.001** |
| **History of falls, n (%)** | **0 (0%)** | **6 (9%)** | **7 (41%)** | **0.002** |
| Cognitive impairment, n (%) | 1 (6%) | 13 (21%) | 7 (44%) | 0.05 |
| **Low mood, n (%)** | **1 (6%)** | **10 (16%)** | **7 (44%)** | **0.02** |
| **Malnutrition, n (%)** | **6 (38%)** | **26 (41%)** | **13 (81%)** | **0.01** |
| **Other holistic interventions, n (%)** | **9 (56%)** | **55 (86%)** | **16 (94%)** | **0.01** |
|  |  |  |  |  |
| HbA1c at GOLD clinic (mmol/L), median [Q1;Q3] | 50.5 [48.0;55.2] | 51.0 [47.0;56.0] | 51.0 [48.0;60.0] | 0.94 |
| Random CBG in GOLD clinic (mmol/L), median [Q1;Q3] | 8.00 [6.00;9.07] | 7.90 [6.80;10.40] | 8.70 [7.40;9.72] | 0.67 |
| All CBG readings (mmol/L), median [Q1;Q3] | 9.36 [8.08;11.36] | 8.54 [7.73;9.97] | 9.98 [7.84;10.96] | 0.32 |
| Glucose AM readings (mmol/L), median [Q1;Q3] | 7.71 [7.33;8.72] | 7.40 [6.93;8.21] | 8.32 [6.86;9.53] | 0.56 |
| Glucose PM readings (mmol/L), median [Q1;Q3] | 11.70 [9.91;12.20] | 10.07 [8.46;10.80] | 11.05 [8.83;12.53] | 0.14 |
| Highest glucose reading (mmol/L), mean ± SD | 17.34 ± 5.24 | 15.67 ± 4.60 | 17.07 ± 5.86 | 0.44 |
|  |  |  |  |  |
| Number of cancer treatment cycles, median [Q1;Q3] | 3 [1;4] | 3 [1;5] | 3 [1;6] | 0.58 |
| Any change in cancer treatment plan during CBG monitoring, n (%) | 7 (47%) | 30 (49%) | 8 (47%) | 0.98 |
| Any change in steroid regimens during CBG monitoring, n (%) | 3 (20%) | 13 (22%) | 5 (29%) | 0.76 |
|  |  |  |  |  |
| New diabetes medication intervention needed, n (%) | 6 (43%) | 19 (33%) | 5 (33%) | 0.75 |

Abbreviations: ADL=activities in daily living; AM=morning time; CBG=capillary blood glucose; CFS=clinical frailty scale; GOLD=geriatric oncology liaison development; PM=evening time; SD=standard deviation.

# **Supplementary data 3:** Association among clinical and biological characteristics and steroid-induced hyperglycaemia in the GOLD patients who underwent a comprehensive geriatric assessment (n=102)

| **Variables** | **Steroid-induced hyperglycaemia (n=30)** | **No Steroid-induced hyperglycaemia (n=62)** | **P-value** |
| --- | --- | --- | --- |
| Age in years, mean ± SD | 73.9 ± 5.6 | 75.5 ± 5.9 | 0.23 |
| **Sex female, n (%)** | **19 (63%)** | **20 (32%)** | **0.005** |
| Metastatic status, n (%) | 11 (37%) | 24 (41%) | 0.67 |
| Pre-existing diabetes, n (%) | 26 (87%) | 45 (73%) | 0.13 |
| CFS, median [Q1;Q3] | 3.00 [3.00;4.00] | 3.00 [3.00;4.00] | 1.00 |
| Social isolation, n (%) | 1 (3%) | 3 (5%) | 1.00 |
| Dependency in ADL, n (%) | 3 (10%) | 9 (15%) | 0.74 |
| Timed get up and go test (sec), median [Q1;Q3] | 12.6 [10.3;16.3] | 13.9 [11.2;19.9] | 0.26 |
| History of falls, n (%) | 5 (17%) | 8 (13%) | 0.75 |
| **Cognitive impairment, n (%)** | **2 (7%)** | **16 (26%)** | **0.04** |
| Low mood, n (%) | 5 (17%) | 12 (20%) | 0.78 |
| Malnutrition, n (%) | 13 (45%) | 28 (46%) | 0.92 |
| Other holistic interventions, n (%) | 24 (80%) | 50 (82%) | 0.82 |
|  |  |  |  |
| HbA1c at GOLD clinic (mmol/mol), median [Q1;Q3] | 54 [48;60] | 50 [45;55] | 0.05 |
| HbA1c at GOLD clinic (%), median [Q1;Q3] | 7.1 [6.6;7.6] | 6.7 [6.3;7.2] | 0.05 |
| Random CBG in GOLD clinic (mmol/L), median [Q1;Q3] | 8.90 [6.90;11.00] | 7.90 [6.50;9.75] | 0.16 |
| **All CBG readings (mmol/L), mean ± SD** | **10.85 ± 2.03** | **8.28 ± 1.18** | **<0.001** |
| **Glucose AM readings (mmol/L), mean ± SD** | **9.21 ± 2.28** | **7.41 ± 1.20** | **<0.001** |
| **Glucose PM readings (mmol/L), mean ± SD** | **12.61 ± 2.61** | **9.28 ± 1.57** | **<0.001** |
| **Highest glucose reading (mmol/L), mean ± SD** | **20.68 ± 4.21** | **13.47 ± 2.96** | **<0.001** |
|  |  |  |  |
| Number of cancer treatment cycles, median [Q1;Q3] | 4 [2;6] | 3 [1;5] | 0.07 |
| Any change in cancer treatment plan during CBG monitoring, n (%) | 17 (57%) | 32 (54%) | 0.83 |
| **Any change in steroid regimens during CBG monitoring, n (%)** | **14 (47%)** | **9 (15%)** | **0.001** |

Abbreviations: ADL=activities in daily living; AM=morning time; CBG=capillary blood glucose; CFS=clinical frailty scale; EGS=comprehensive geriatric assessment; GOLD=geriatric oncology liaison development; PM=evening time; SD=standard deviation.

Restricted to patients who underwent a comprehensive geriatric assessment (n=102).

**Supplementary data 4**: Univariate analysis of overall survival in the GOLD patients (n=126)

| Variables | Univariate analysis  HR (95% CI), p-value |
| --- | --- |
| Age in years  Gender (Male vs Female)  **Metastatic status (Y vs N)**  Pre-existing diabetes (Y vs N)  CFS (per unit)  **Social isolation (Y vs N)**  Dependency in ADL (Y vs N)  Timed Get up and Go (per second)  History of falls (Y vs N)  Cognitive impairment (Y vs N)  Low mood (Y vs N)  **Malnutrition (Y vs N)**  Geriatric interventions other than DP (Y vs N)  HbA1c at GOLD clinic (per mmol/L)  Random CBG in GOLD clinic (per unit)  CBG readings (per unit)  CBG AM readings (per unit)  CBG PM readings (per unit)  Highest CBG reading (per unit)  Diabetes medication intervention | 1.01 (0.96-1.07), p =0.59  1.78 (0.86-3.69), p = 0.12  **2.43 (1.23-4.8), p =0.01**  0.80 (0.41-1.57), p =0.51  1.28 (0.97-1.69), p = 0.08  **3.34 (1.15-9.68), p = 0.03**  1.71 (0.71-4.71), p = 0.23  1.04 (0.98-1.09), p = 0.17  1.78 (0.72-4.37), p = 0.21  0.62 (0.24-1.59), p = 0.32  1.11 (0.46-2.69), p = 0.81  **3.00 (1.45-6.20), p = 0.001**  2.53 (0.88-7.30), p = 0.09  1.02 (0.99-1.05), p = 0.12  1.05 (0.95-1.15), p = 0.35  1.12 (0.92-1.37), p = 0.27  1.10 (0.90-1.35), p = 0.34  1.01 (0.84-1.20), p = 0.92  0.98 (0.89-1.07), p =0.60  1.22 (0.57-2.61), p = 0.60 |

Abbreviations: ADL= activities in daily living; AM= morning time; CBG=Capillary Blood Glucose; CFS=clinical frailty scale; CI= confidence interval; DP= diabetes pathway; GOLD=geriatric oncology liaison development; HR=hazard ratio; PM=evening time
